# *TLR7* polymorphisms are associated with COVID-19 susceptibility and severity

**DOI:** 10.3389/fimmu.2026.1837208

**Published:** 2026-05-28

**Authors:** Edyta Paradowska, Katarzyna D. Kania, Dariusz Jarych, Damian Mikulski, Elżbieta Jabłonowska, Daria A. Haręża, Kamila Wójcik-Cichy

**Affiliations:** 1 Laboratory of Virology, Institute of Medical Biology of the Polish Academy of Sciences, Lodz, Poland; 2 Department of Biostatistics and Translational Medicine, Medical University of Lodz, Lodz, Poland; 3 Clinic of Infectious Diseases and Hepatology, Medical University of Lodz, Lodz, Poland; 4 BioMedChem Doctoral School of the University of Lodz and Lodz Institutes of the Polish Academy of Sciences, Lodz, Poland

**Keywords:** COVID-19, cytokine, polymorphism, RLR, SARS-CoV-2, TLR

## Abstract

**Objectives:**

SARS-CoV-2 infection causes an innate immune response that is activated through pattern-recognition receptors (PRRs), including Toll-like receptors (TLRs) and retinoic acid-inducible gene I (RIG-I)-like receptors (RLRs). Endosomal TLR7/8 detects viral ssRNA, while TLR3 also recognizes dsRNA formed during viral replication. Both RIG-I and MDA5 recognize SARS-CoV-2 RNA in the cytoplasm of infected cells. PRR pathways recruit essential downstream adapter proteins to induce type I interferons (IFNs) and inflammatory cytokines. While genetic variations in host *PRRs* may influence SARS-CoV-2 infection, immune response, and COVID-19 severity, their specific role in triggering these processes remains unclear. This study analyzes the frequency of specific polymorphisms within *PRR* genes in COVID-19 patients to evaluate their impact on disease severity.

**Methods:**

We genotyped ten *PRR* polymorphisms in 261 individuals, including 166 patients hospitalized for COVID-19, and evaluated their associations with clinical parameters and serum cytokine profiles. Single-nucleotide polymorphisms (SNPs) of *TLR3* (rs3775290 and rs3775291), *TLR7* (rs179008, rs3853839, and rs5741880), *TLR8* (rs3764879 and rs3764880), *IFIH1* rs1990760, and *DDX58* rs73479410 were genotyped using qPCR allelic discrimination, while *TLR3* rs3775296 was analyzed by PCR-RFLP. Cytokine concentrations were quantified using MILLIPLEX Magnetic Bead Panels and Luminex xMAP technology.

**Results:**

In this case-control study, the recessive G/G genotype of the *TLR7* rs3853839 SNP was associated with an increased risk of hospitalization. The presence of at least one recessive T allele for the *TLR7* rs179008 SNP was associated with lower cytokine levels (IP-10, IL-10, TNF-α) and a decreased risk of severe symptoms in hospitalized patients.

**Conclusions:**

Our findings suggest that the *TLR7* rs3853839 SNP may represent a genetic risk factor for severe COVID-19, whereas the missense *TLR7* rs179008 SNP (Q11L) may contribute to less severe symptoms among these patients.

## 1 Introduction

Severe acute respiratory syndrome coronavirus 2 (SARS-CoV-2) is a single-stranded positive-sense RNA (+ssRNA) pathogen responsible for the coronavirus disease (COVID-19). The virus belongs to the family *Coronaviridae* in the *Nidovirales* order and was classified as a member of the *Betacoronavirus* genus (1). The clinical manifestations of COVID-19 are highly variable across individuals and can range from asymptomatic infection to critical illness. The common symptoms of disease include fever, cough, myalgia or fatigue, headache, and gastrointestinal symptoms such as nausea, vomiting, and diarrhea (2, 3). Data from 2020 demonstrated that approximately 80% of symptomatic patients developed mild (40%) or moderate (40%) disease (4, 5). Approximately 15% of SARS-CoV-2-infected individuals developed severe disease, which can progress to severe pneumonia that requires oxygen support. Approximately 5% of patients present with critical disease with complications such as respiratory failure, acute respiratory distress syndrome (ARDS), sepsis, multiorgan dysfunction, and death (4, 5). Compared with Delta-variant periods, decreased pathogenicity and increased transmissibility were observed during the Omicron-variant period (6, 7). In addition to the virulence of the virus strain, individual risk factors such as older age, male sex, Asian and Black ethnic backgrounds, and comorbidities, including diabetes, hypertension, immunosuppression, cancer, cardiovascular and respiratory diseases, chronic kidney disease, and obesity, have been reported to increase COVID-19 severity (8–11). However, these factors only partially explain the interindividual variability in disease severity. The role of host genetic factors in susceptibility to infection and COVID-19 severity has been of significant interest (12–16). The identified *loci* associated with COVID-19 susceptibility and severity include genes involved in three pathways: viral entry, entry defense in airway mucus, and type I interferon (IFN-I) response (15). Mutations in genes involved in innate immune pathways that impair IFN-I responses have been linked to severe disease (17, 18). Dysregulation of the immune response, which results in the suppression of the IFN-I response and overproduction of inflammatory cytokines, is observed in severe and critical forms of COVID-19 (7, 19).

SARS-CoV-2-associated ssRNA and dsRNA transcription intermediates formed during viral replication are sensed by pattern-recognition receptors (PRRs) within endosomes, including Toll-like receptors (TLRs), and by cytoplasmic RNA sensors, which promote the synthesis of type I (20–22) and type I/III IFNs (23, 24), respectively. Among TLRs, TLR3, TLR7, and TLR8 are located on the endosomal membrane and can recognize viral RNA. TLR3 recognizes viral double-stranded (ds) RNA, while TLR7 and TLR8 bind G/U-rich viral ssRNA. TLR signaling pathways recruit important downstream adaptor proteins, such as, MyD88 and TRIF, leading to the activation of transcription factors, including interferon regulatory factors (IRFs) and NF-κB, and subsequent induction of IFN-I and inflammatory cytokines. SARS-CoV-2 ssRNA can bind TLR7 and TLR8, while viral dsRNA generated during replication can bind to TLR3 in host cells, including macrophages and dendritic cells (25). TLR7 and TLR8 were shown to induce proinflammatory cytokines in response to SARS-CoV-2 RNA (26, 27). Retinoic acid-inducible gene I (RIG-I)-like receptors (RLRs), such as RIG-I and interferon-induced helicase C domain-containing protein 1 (IFIH1, known as melanoma differentiation-associated protein 5, MDA5), are cytosolic helicases that mediate an effective immune response, including the transcriptional induction of genes encoding type I IFNs and proinflammatory cytokines (28). RIG-I and MDA5 are encoded by the *DDX58* and *IFIH1* genes, respectively, which are responsible for recognizing viral ssRNA and dsRNA and are crucial for antiviral defense (29). SARS-CoV-2 replicates in lung epithelial cells and induces an innate immune response through the activation of RIG-I and MDA5 (23).

Single-nucleotide polymorphisms (SNPs) in genes that are linked to the IFN-I pathway have been associated with susceptibility to and severity of COVID-19. A significant association between *TLR7* SNPs and COVID-19 susceptibility and severity has been reported (30–32). Individuals with rare deleterious variants in *TLR7* are at increased risk of severe COVID-19 (20, 22, 33, 34). The GG genotype of *TLR7* rs3853839 was found to be a potential risk factor for COVID-19 infection in individuals without previous comorbidities (31). Moreover, this genotype was associated with higher disease severity (32). Associations have also been reported between specific *TLR3* genetic variants and both the incidence and severity of COVID-19 (35, 36). The minor allele frequency of *TLR3* rs3775291 was positively correlated with COVID-19 cases and associated mortality (35). In addition, the *DDX58* rs10813831 (37, 38) and *IFIH1* rs1990760 polymorphisms (39–41) were associated with COVID-19 outcomes. Given the population-specific genetic diversity of *PRRs* and inconsistencies in observed findings, further confirmatory and exploratory studies are required.

It was hypothesized that polymorphisms in the *PRR* genes may modulate the host response and influence the clinical outcome of COVID-19. Hence, the relevance of *PRR* SNPs was investigated in a cohort of 261 adults, which comprised 166 hospitalized patients diagnosed with COVID-19. We determined the distribution of the *TLR3* (rs3775290, rs3775291, and rs3775296), *TLR7* (rs179008, rs3853839, and rs5741880), *TLR8* (rs3764879 and rs3764880), *IFIH1* (rs1990760), and *DDX58* (rs73479410) genotypes and investigated their associations with symptoms and cytokine expression in patients hospitalized for COVID-19. The selection of *PRR* SNPs was based on their possible functional effect and the existence of previous associations with COVID-19 susceptibility or severity (30–32, 35, 36, 38–41).

## 2 Materials and methods

### 2.1 Study group

The study included 261 adults, divided into two groups: 166 patients hospitalized for COVID-19 and 95 healthy volunteers from the general population, who served as age- and sex-matched controls. Patients with COVID-19 were enrolled between January–June 2021 and January–June 2022 at the Clinic of Infectious Diseases and Hepatology, Medical University of Lodz, covering the third (Alpha-dominant) and fifth (Omicron) SARS‐CoV‐2 waves in Poland (42). Hospitalized patients with a registered positive SARS-CoV-2 test were categorized into severity index categories 1–10 based on the WHO Clinical Progression Scale (43, 44). Mild disease was defined as the presence of typical COVID-19 symptoms (e.g., fever, cough, fatigue, myalgias, headache, sore throat, nasal congestion, nausea, vomiting, diarrhea, anosmia, or ageusia) without evidence of viral pneumonia or hypoxemia (score ≤ 3). Patients with moderate disease had clinical signs of pneumonia (e.g., fever, cough, dyspnea, and fast breathing) but no signs of severe pneumonia (scores 4 or 5); moderate disease is subdivided into two categories: “hospitalized; no oxygen therapy” (score 4) and “hospitalized; oxygen by mask or nasal prongs” (score 5) (43). Patients with severe disease (scores 6–9) had clinical indications of pneumonia (fever, cough, or dyspnea) plus one of the following: respiratory rate > 30 breaths/min, severe respiratory distress, or SpO_2_ < 90% on room air. This WHO category is subdivided into four categories: “hospitalized; oxygen by non-invasive ventilation or high flow” (score 6), “intubation and mechanical ventilation, pO_2_/FiO_2_ ≥150 or SpO_2_/FiO_2_ ≥200” (score 7), “mechanical ventilation pO_2_/FiO_2_ <150 or vasopressors” (score 8), and “mechanical ventilation pO_2_/FiO_2_ <150 and vasopressors, dialysis, or extracorporeal membrane oxygenation (ECMO)” (score 9). Critical illness included patients who met the criteria for acute respiratory distress syndrome (ARDS), sepsis or septic shock, or who required life-sustaining interventions such as invasive/noninvasive mechanical ventilation or vasopressor therapy, or acute thrombosis, as clinically assessed, and who needed intensive management. SARS-CoV-2 infection was confirmed by real-time RT-PCR in the nasal swabs of hospitalized patients. In almost all patients (164/166; 98.80%), PCR confirmation occurred within 14 days before sample collection. The period from the detection of SARS-CoV-2 RNA exceeded two weeks in two patients. Peripheral blood and serum samples were collected during hospitalization and stored at -80 °C. Whole blood and serum aliquots were thawed immediately before the genomic DNA isolation and Luminex assays, respectively. The demographic and clinical characteristics of patients with COVID-19 are summarized in **Table 1**.

**Table 1:** Study group characteristics.

Demographic characteristics	n (%)
Total No.	166 (100)
Mean ± SD age, years	59.7 ± 14.73
Median (IQR), years	63 (50.5 – 70.0)
Gender number	
Female	72 (43.37)
Male	94 (56.63)
Time of infection	
January – June 2021	143 (86.14)
January – June 2022	23 (13.86)
Clinical characteristics	
Symptoms/signs	
pneumonia	156 (93.98)
fever	141 (84.94)
cough	134 (80.72)
dyspnea	115 (69.28)
gastrointestinal symptoms	36 (21.69)
dimer growth	23 (13.86)
smell and/or taste disorders	19 (11.44)
neurological symptoms	19 (11.44)
pulmonary embolism	7 (4.22)
Clinical and laboratory assessments	
O_2_ saturation	89%, 84-94%
CRP	55.41 mg/L, 16.57 – 113.47 mg/L
D-dimers	888.5 ng/mL, 581–1479 ng/mL
WHO severity scale	
4	41 (24.70)
5	107 (64.46)
6	9 (5.42)
7	0 (0)
8	1 (0.60)
9 10	0 (0) 7 (4.22)
No data	1 (0.60)
Hospitalization	
Yes	166 (100)
No	0 (0)
Comorbidities	
No	31 (18.67)
Yes	134 (80.72)
hypertension	67 (50.00)
diabetes	23 (17.16)
obesity	21 (15.67)
CAD	20 (14.93)
asthma	16 (11.94)
cancer	14 (10.45)
COPD	12 (8.96)
circulatory failure	12 (8.96)
AFL or AFib	9 (6.72)
No data	1 (0.60)
CCI	
0-1	54 (32.53)
2-3	58 (34.94)
4-5	34 (20.48)
≥6	15 (9.04)
Vaccine before infection	
No	148 (89.16)
Yes	18 (10.84)
1 dose	10 (55.56)
2 doses	5 (27.78)
3 doses	3 (16.67)

Data are presented as frequencies with corresponding percentages. For continuous variables, the median with interquartile range is reported. n, number of patients (%); IQR, interquartile range; CRP, C-reactive protein; CAD, coronary artery disease; COPD, chronic obstructive pulmonary disease; AFL, atrial flutter; AFib, atrial fibrillation; CCI, Charlson Comorbidity Index.

### 2.2 PRR genotyping

Genomic DNA was extracted from the thawed whole-blood samples collected in EDTA tubes using the QIAamp DNA Blood Mini Kit on a QIAcube automated workstation (Qiagen, Hilden, Germany) according to the manufacturer’s instructions. DNA concentration and purity were assessed using a NanoDrop 2000c UV–Vis Spectrophotometer (Thermo Fisher Scientific, Waltham, MA, USA). A total of ten SNPs within the *PRR* genes, including *TLR3*, *TLR7*, *TLR8*, *IFIH1*, and *DDX58*, were analyzed. Genotyping of *TLR3* (rs3775290 C>T; F459F and rs3775291 C>T; L412F), *TLR7* (rs179008 A>T; Q11L, rs3853839 C>G, and rs5741880 G>T), *TLR8* (rs3764879 C>G and rs3764880 A>G), *IFIH1* (rs1990760 C>T; T946A), and *DDX58* (rs73479410 G>A) was performed using TaqMan™ SNP Genotyping Assays and TaqMan Genotyping Master Mix (Thermo Fisher Scientific). Real-time PCR was conducted using the QuantStudio 5 system (Thermo Fisher Scientific). *TLR3* rs3775296 C>A was analyzed using the polymerase chain reaction-restriction fragment length polymorphism (PCR-RFLP) method as described previously (45). Each reaction mixture consisted of 0.5 μg (5 μL) of template DNA, 5 μL of 10 × DreamTaq™ Buffer (ThermoFisher Scientific, Vilnius, Lithuania), 5 μL of 2.5 mM dNTPs, 0.5 μL of each gene-specific primer (100 pmol/μL; Genomed, Warsaw, Poland), and 0.25 μL of DreamTaq™ Polymerase (5 U/μL; ThermoFisher Scientific), adjusted with nuclease-free water to a final volume of 50 μL. The thermal profile included an initial denaturation at 95 °C for 15 min, followed by 35 cycles of 94 °C for 30 s, 62 °C for 30 s, and 72 °C for 30 s, with a final extension step at 72 °C for 7 min. PCR reactions were performed in a Biometra TAdvanced thermal cycler (Analytik Jena GmbH, Göttingen, Germany). The PCR products were digested overnight at 37 °C with 1 µL of *Mbo*II restriction enzyme (5 U/μL; Thermo Fisher Scientific). The digested fragments were analyzed with the QIAxcel DNA Screening Kit (Qiagen) and the QIAxcel capillary electrophoresis system (Qiagen). Fragment sizes were determined using the QX DNA Size Marker 50–800 bp and BioCalculator software (Qiagen).

### 2.3 Multiplex immunoassay

Serum samples from patients hospitalized for COVID-19 were analyzed using bead-based multiplex immunoassays based on Luminex xMAP technology (Luminex Corp., Austin, TX, USA) with MILLIPLEX^®^ kits (Merck KGaA, Darmstadt, Germany). The samples were analyzed in duplicate using the MILLIPLEX^®^ Human Cytokine/Chemokine/Growth Factor Panel A (16 analytes, including IFN-β, IFN-γ, IL-1RA, IL-4, IL-6, IL-8/CXCL8, IL-10, IL-18, IL-28A/IFN-λ2, IP-10/CXCL10, MCP-1, MCP-3, TNF-α, TNF-β, M-CSF, and MIP-1β), according to the manufacturer’s recommendations. Standard curves, blanks, and kit quality controls were included on each plate. Briefly, 25 µL of each undiluted serum sample was mixed with 25 µL of antibody-linked magnetic beads in a 96-well plate and incubated for 2 hours at room temperature with orbital shaking (600 rpm). All room-temperature incubation steps (with the detection antibody and streptavidin-PE) were performed on an orbital shaker at 600 rpm. The plates were subsequently washed three times with wash buffer using a magnetic plate washer. Following a 1-hour incubation at room temperature with a biotinylated detection antibody under continuous shaking (~600 rpm), streptavidin-PE was added for 30 minutes with continued shaking. The plates were subsequently washed three times as described above, after which the beads were resuspended in 150 µL of MAXPIX Drive Fluid (Luminex Corp.). Data acquisition was performed using a MAGPIX System (Luminex Corp.) and the xPONENT for MAGPIX v. 4.2 software (Luminex Corp.). A minimum of 50 beads per analyte per well were collected. The cytokine/chemokine/growth factor concentrations were reported as pg/mL, in accordance with the manufacturer’s recommendations for serum samples. Analyte concentrations were calculated from seven-point standard curves using a five-parameter logistic regression model implemented in Belysa (Merck) analysis software. Replicate pairs with a coefficient of variation (CV) >15% (intra-assay %CV) were re-assayed or excluded.

### 2.4 Statistical analysis

Hardy-Weinberg equilibrium (HWE) and single-SNP analysis were performed using SNPStats software, https://snpstats.net (46, 47). The distribution of genotypes and alleles was examined using the chi-square (χ²) test with Yates’ correction. Multiple-SNP analyses, including linkage disequilibrium (LD) and haplotype analyses, were performed using Haploview software version 4.2 (Broad Institute, Cambridge, MA, USA) (48, 49). Odds ratios (ORs) and 95% confidence intervals (95% CIs) with both unadjusted and adjusted multivariate models were calculated to examine the association between the *PRR* SNPs and COVID-19. Linkage disequilibrium (LD) analysis was performed for each pair of polymorphisms using D’ and the correlation coefficient r^2^, indicating the amount of LD between two genetic loci. Haplotype blocks were defined using the Solid Spine of LD algorithm. Statistical associations were evaluated by comparing haplotype frequencies between cases and controls using Pearson’s χ² test. ORs were derived from 2×2 contingency tables; notably, ORs were not calculated for haplotypes that were absent in either the case or control groups.

The distribution of continuous variables was assessed using the Shapiro-Wilk test. Data are presented as frequencies and percentages, whereas continuous variables are expressed as medians and interquartile ranges (IQRs) due to the lack of a normal distribution. To evaluate associations between the genotypes of the analyzed SNPs and groups categorized by COVID-19 severity, Pearson’s χ² test was used. Where necessary, appropriate corrections were applied: Yates’s correction for continuity or Fisher’s exact test. In general, if any of the expected frequencies in a 2×2 contingency table were less than 15, Yates’s correction was used; if they were less than 5, Fisher’s exact test was applied.

In the next step, univariate and multivariate logistic regression models were constructed to identify predictors of severe COVID-19. The goodness of fit of the final multivariate model was assessed using the Hosmer-Lemeshow test, where a *p*-value above 0.05 indicated a good fit. The receiver operating characteristic (ROC) curve and area under the ROC curve (AUC) were used to visualize the discriminative ability of the final model, and Youden’s method was applied to determine the optimal sensitivity and specificity cutoff points. When cytokine protein levels were analyzed, missing values due to measurements below the limit of detection (LOD) were imputed using half of the LOD value. If the kit manufacturer did not provide an LOD value, missing values (recorded as 0) were replaced with half of the minimum value observed in the analyzed cohort. However, five cytokines were excluded from the analysis because of a high proportion (>50%) of missing values or measurements below the LOD: MIP-1α, IL-1β, IL-29, IL-35, and IFN-α2. Exploratory data analysis was conducted using heatmap visualization, which was based on log_2_-transformed values. Hierarchical clustering analysis was performed using the Euclidean distance measure and the complete linkage method. The heatmap was generated using Morpheus (https://software.broadinstitute.org/morpheus). Due to the nonnormal distribution of protein levels, comparisons between groups were made using the non-parametric Mann-Whitney U test. Due to the exploratory nature of our cytokine and other protein comparisons, with the primary aim of identifying potential trends within a limited sample size, no correction for multiple comparisons was applied. However, we acknowledge the associated risk of false-positive results. The statistical analyses were conducted using Statistica 13.3 (TIBCO, Palo Alto, CA, USA) and GraphPad Prism 9.00 (San Diego, CA, USA).

*p*-values less than 0.05 were considered to indicate statistical significance. Bonferroni correction (*p_B_*) of the significance level was applied exclusively to the SNP analyses to account for multiple comparisons. The significance level was applied for eight multiple comparisons; the significance level for *p_B_* was 0.00625 instead of the standard 0.05.

## 3 Results

### 3.1 Study group characteristics

One hundred sixty-six patients with COVID-19 (median age: 63 years; range 23–90 years; IQR: 50.5–70.0 years) and 95 sex- and age-matched healthy volunteers were examined. The majority of patients with COVID-19 were older than 55 years (108/166, 65.06%), with slightly more male participants (55.42%). All patients in the study cohort required hospitalization, with the majority (63.86%) needing oxygen therapy. In all patients with COVID-19, infection with SARS-CoV-2 was confirmed by real-time RT-PCR. Approximately 86% of the cases were collected during the Alpha SARS‐CoV‐2 wave, while the remaining patients were infected with SARS-CoV-2 during the Omicron wave in Poland. Ten patients (6.02%) had severe disease according to the WHO Clinical Progression Scale (scores 6–9), and ultimately, seven patients (4.22%) died due to COVID-19 (score 10). Moderate disease (scores 4 or 5) was diagnosed in 148/166 (89.16%) patients. The median oxygen saturation at admission was 89% (IQR: 84–94%), while high-flow nasal oxygen therapy (HFNOT) was used in 17 patients (10.24%). Four symptoms were reported in more than 50% of the patients: pneumonia (93.98%), fever (84.94%), cough (80.72%), and dyspnea (69.28%). More than three-quarters of the patients (134/166, 80.72%) reported preexisting comorbidities. The most common comorbidities were: hypertension (50%), diabetes (17.16%), obesity (15.67%), coronary artery disease (CAD, 14.93%), asthma (11.94%), and cancer (10.45%). Other comorbidities were present in fewer than 10% of study participants. The median CRP concentration was 55.41 mg/L (IQR: 16.57–113.47 mg/L). Due to the period of SARS-CoV-2 infection (2021/2022), the vast majority of participants were either unvaccinated or had received fewer than three doses of the COVID-19 vaccine (163/166, 98.19%). The detailed study characteristics are provided in **Table 1**.

### 3.2 The homozygous GG genotype and G allele of *TLR7* rs3853839 polymorphism occur more frequently in patients with COVID-19 than in healthy controls

The *TLR3* (rs3775290, rs3775291, and rs3775296), *TLR7* (rs179008, rs3853839, and rs5741880), *TLR8* (rs3764879 and rs3764880), *IFIH1* (rs1990760), and *DDX58* (rs73479410) SNPs were genotyped in patients with COVID-19 and healthy volunteers (**Table 2**). With respect to the *TLR7* rs3853839 SNP, the homozygous recessive GG genotype was detected more frequently in patients with COVID-19 than in healthy controls (13.0% vs. 3.2%, *p* = 0.020; **Table 2**). Consequently, the G allele of this SNP was detected more frequently in the study group than in the control group (20.2% vs. 11.1%, *p* = 0.013; **Table 2**). No significant sex-related differences in the distribution of rs3853839 genotypes were observed among patients with COVID-19 (*p* > 0.05). The recessive alleles for the *TLR3* rs3775296, *TLR7* rs3853839 SNPs, and both *TLR8* SNPs were detected more frequently in women hospitalized for COVID-19 than in the control group (*p* < 0.05; **Supplementary Table 1**). However, because the sample size of female cases in the study group was relatively small, these results should be interpreted with caution. No other significant case–control differences in the distribution of other SNPs were found.

**Table 2:** Frequencies of *TLR3*, *TLR7*, *TLR8*, *IFIH1*, and *DDX58* SNPs genotypes and alleles in healthy volunteers (controls) and patients with COVID-19 (cases).

Gene	SNP ID	Genotype/allele	Genotype/allele frequencies, n (%)		*p*-value
			Controls	Cases	
*TLR3*	rs3775290	CC	46 (48.4)	56 (45.2)	0.817
		CT	40 (42.1)	50 (40.3)	0.899
		TT	9 (9.5)	18 (14.5)	0.359
		C	132 (69.5)	162 (65.3)	0.416
		T	58 (30.5)	86 (34.7)	
	rs3775291	CC	49 (51.6)	83 (50.9)	0.919
		CT	36 (37.9)	64 (39.3)	0.932
		TT	10 (10.5)	16 (9.8)	0.855
		C	134 (70.5)	230 (70.6)	0.995
		T	56 (29.5)	96 (29.4)	
	rs3775296	CC	60 (71.4)	71 (59.7)	0.115
		CA	24 (28.6)	43 (36.1)	0.328
		AA	0 (0)	5 (4.2)	0.149
		C	144 (85.7)	185 (77.7)	0.058
		A	24 (14.3)	53 (22.3)	
*TLR7*	rs179008	AA	56 (59.6)	113 (68.9)	0.167
		AT	25 (26.6)	31 (18.9)	0.199
		TT	13 (13.8)	20 (12.2)	0.853
		A	137 (73)	257 (78)	0.193
		T	51 (27)	71 (22)	
	rs3853839	CC	77 (81.0)	95 (72.5)	0.184
		CG	15 (15.8)	19 (14.5)	0.937
		GG	3 (3.2)	17 (13.0)	**0.020**
		C	169 (88.9)	209 (79.8)	**0.013**
		G	21 (11.1)	53 (20.2)	
	rs5741880	GG	80 (87.9)	90 (93.8)	0.257
		GT	11 (12.1)	6 (6.3)	
		G	171 (94.0)	186 (96.9)	0.269
		T	11 (6.0)	6 (3.1)	
*TLR8*	rs3764879	CC	70 (73.7)	89 (68.5)	0.483
		CG	20 (21.1)	23 (17.7)	0.644
		GG	5 (5.3)	18 (13.8)	0.061
		C	160 (84)	201 (77)	0.090
		G	30 (16)	59 (23)	
	rs3764880	AA	70 (73.7)	92 (68.7)	0.499
		AG	20 (21.1)	26 (19.4)	0.889
		GG	5 (5.3)	16 (11.9)	0.135
		A	160 (84)	210 (78)	0.148
		G	30 (16)	58 (22)	
*IFIH1*	rs1990760	CC	10 (10.5)	21 (12.7)	0.742
		CT	40 (42.1)	75 (45.5)	0.694
		TT	45 (47.4)	69 (41.8)	0.460
		C	60 (32)	117 (35)	0.422
		T	130 (68)	213 (65)	
*DDX58*	rs73479410	GG	56 (59.0)	103 (62.4)	0.673
		AG	32 (33.7)	55 (33.3)	0.954
		AA	7 (7.4)	7 (4.2)	0.429
		G	144 (76)	261 (79)	0.445
		A	46 (24)	69 (21)	

n, number of cases; *p*, Chi-square (χ²) test with Yates’ correction. Significant *p*-values are highlighted in bold.

### 3.3 The GG genotype of *TLR7* rs3853839 polymorphism is associated with an increased risk of COVID-19-related hospitalization

The genotype and allele frequencies for all the evaluated *TLR3*, *TLR8*, *IFIH1*, and *DDX58* polymorphisms, as well as for *TLR7* (rs3853839), were in Hardy-Weinberg equilibrium (HWE) in the control group. In contrast, *TLR7* rs179008 deviated from HWE in controls (*p* ≤ 0.050) and was therefore excluded from subsequent analysis. In the group of patients with COVID-19, deviations from HWE were observed for the *TLR7* rs3853839, *TLR8* rs3764879, and *TLR8* rs3764880 SNPs, whereas all other polymorphisms remained in equilibrium. Given that deviations from HWE in affected individuals do not preclude association testing, these SNPs were retained for further analyses. Only *TLR* and *RLR* SNPs with a minor allele frequency (MAF) > 0.01 in the control group were included in the association analysis. Because the intronic *TLR7* rs5741880 SNP did not meet these conditions, it was excluded from both the single-marker and multi-SNP analyses (**Tables 2**, **3**). Thus, eight polymorphisms were included in the case–control analyses: *TLR3* (rs3775290, rs3775291, and rs3775296), *TLR7* (rs3853839), *TLR8* (rs3764879 and rs3764880), *IFIH1* rs1990760, and *DDX58* rs73479410 (**Table 4**). The homozygous recessive genotypes of *TLR3* rs3775296, *TLR7* rs3853839, and *TLR8* rs3764879 SNPs were associated with an increased risk of moderate or severe COVID-19 (*p* = 0.02, *p* = 0.006, and *p* = 0.03, respectively, in the recessive model; **Table 4**). Among them, the GG genotype of *TLR7* rs3853839 SNP was associated with a fourfold increased risk of COVID-19-related hospitalization (OR 4.57; 95% CI: 1.30–16.08; *p* = 0.006; recessive model). The association remained statistically significant after Bonferroni correction for multiple testing (*p_B_* = 0.00625).

**Table 3:** Selected single-nucleotide polymorphisms in *TLR* and *RLR* genes.

Gene	SNP ID	Alleles	Amino acid change	Function	MAF			
					Polish	ALFA	1000G	gnomAD
*TLR3*	rs3775290	C>T	F459F	synonymous	0.3053	0.2114	0.2812	0.3051
	rs3775291	C>T	L412F	missense	0.2947	0.2931	0.3160	–
	rs3775296	C>A	–	UTR	0.1429	0.1884	0.1785	0.1917
*TLR7*	rs179008	A>T	Q11L	missense	0.2713	0.2139	0.221	0.2215
	rs3853839	C>G	–	UTR	0.1105	0.1615	0.162	0.1760
	rs5741880	G>T	–	intronic	0.0604	0.0999	0.126	0.1009
*TLR8*	rs3764879	C>G	–	intronic	0.1579	0.2400	0.262	0.2491
	rs3764880	A>G	M1V	missense	0.1579	0.2650	0.264	0.2497
*IFIH1*	rs1990760	C>T	A946T	missense	0.6842	0.5991	0.6011	0.6033
*DDX58*	rs73479410	G>A	–	intronic	0.2421	0.2033	0.1927	0.2038

MAF, Minor allele frequency in the Polish and European population (ALFA Project, 1000Genomes_30X Project, gnomAD - Genomes).

**Table 4:** The distribution of allele frequencies of *TLR* and *RLR* SNPs in healthy individuals (controls) and patients hospitalized due to COVID-19 (cases).

Gene SNP	Model	Genotype	Genotype frequencies, n (%)		OR (95% CI)	*p-*value	AIC	BIC
			Controls	Cases				
*TLR3* rs3775290	Codominant	C/C	46 (48.4)	56 (45.2)	1.00	0.52	304.4	314.6
		C/T	40 (42.1)	50 (40.3)	1.03 (0.58-1.82)			
		T/T	9 (9.5)	18 (14.5)	1.64 (0.67-4.00)			
	Dominant	C/C	46 (48.4)	56 (45.2)	1.00	0.63	303.5	310.3
		C/T-T/T	49 (51.6)	68 (54.8)	1.14 (0.67-1.95)			
	Recessive	C/C-C/T	86 (90.5)	106 (85.5)	1.00	0.26	302.5	309.2
		T/T	9 (9.5)	18 (14.5)	1.62 (0.69-3.79)			
	Overdominant	C/C-T/T	55 (57.9)	74 (59.7)	1.00	0.79	303.7	310.5
		C/T	40 (42.1)	50 (40.3)	0.93 (0.54-1.60)			
*TLR3* rs3775291	Codominant	C/C	49 (51.6)	83 (50.9)	1.00	0.97	345.5	356.1
		C/T	36 (37.9)	64 (39.3)	1.05 (0.61-1.80)			
		T/T	10 (10.5)	16 (9.8)	0.94 (0.40-2.24)			
	Dominant	C/C	49 (51.6)	83 (50.9)	1.00	0.92	343.5	350.6
		C/T-T/T	46 (48.4)	80 (49.1)	1.03 (0.62-1.70)			
	Recessive	C/C-C/T	85 (89.5)	147 (90.2)	1.00	0.86	343.5	350.6
		T/T	10 (10.5)	16 (9.8)	0.93 (0.40-2.13)			
	Overdominant	C/C-T/T	59 (62.1)	99 (60.7)	1.00	0.83	343.5	350.6
		C/T	36 (37.9)	64 (39.3)	1.06 (0.63-1.78)			
*TLR3* rs3775296	Codominant	C/C	60 (71.4)	71 (59.7)	1.00	0.027	274.1	284
		C/A	24 (28.6)	43 (36.1)	1.51 (0.83-2.78)			
		A/A	0 (0)	5 (4.2)	NA (0.00-NA)			
	Dominant	C/C	60 (71.4)	71 (59.7)	1.00	0.082	276.3	283
		C/A-A/A	24 (28.6)	48 (40.3)	1.69 (0.93-3.08)			
	Recessive	C/C-C/A	84 (100)	114 (95.8)	1.00	0.02	273.9	280.5
		A/A	0 (0)	5 (4.2)	NA (0.00-NA)			
	Overdominant	C/C-A/A	60 (71.4)	76 (63.9)	1.00	0.26	278.1	284.7
		C/A	24 (28.6)	43 (36.1)	1.41 (0.77-2.59)			
*TLR7* rs3853839	Codominant	C/C	77 (81.0)	95 (72.5)	1.00	0.024	306.1	316.4
		C/G	15 (15.8)	19 (14.5)	1.03 (0.49-2.15)			
		G/G	3 (3.2)	17 (13.0)	4.59 (1.30-16.25)			
	Dominant	C/C	77 (81.0)	95 (72.5)	1.00	0.13	309.3	316.1
		C/G-G/G	18 (18.9)	36 (27.5)	1.62 (0.85-3.08)			
	Recessive	C/C-C/G	92 (96.8)	114 (87.0)	1.00	**0.006**	304.1	311
		G/G	3 (3.2)	17 (13.0)	4.57 (1.30-16.08)			
	Overdominant	C/C-G/G	80 (84.2)	112 (85.5)	1.00	0.79	311.5	318.3
		C/G	15 (15.8)	19 (14.5)	0.90 (0.43-1.89)			
*TLR8* rs3764879	Codominant	C/C	70 (73.7)	89 (68.5)	1.00	0.09	307.6	317.9
		C/G	20 (21.1)	23 (17.7)	0.90 (0.46-1.78)			
		G/G	5 (5.3)	18 (13.8)	2.83 (1.00-8.00)			
	Dominant	C/C	70 (73.7)	89 (68.5)	1.00	0.39	309.7	316.6
		C/G-G/G	25 (26.3)	41 (31.5)	1.29 (0.72-2.32)			
	Recessive	C/C-C/G	90 (94.7)	112 (86.2)	1.00	0.03	305.7	312.5
		G/G	5 (5.3)	18 (13.8)	2.89 (1.03-8.09)			
	Overdominant	C/C-G/G	75 (79.0)	107 (82.3)	1.00	0.53	310.1	316.9
		C/G	20 (21.1)	23 (17.7)	0.81 (0.41-1.57)			
*TLR8* rs3764880	Codominant	A/A	70 (73.7)	92 (68.7)	1.00	0.21	313.6	323.9
		A/G	20 (21.1)	26 (19.4)	0.99 (0.51-1.92)			
		G/G	5 (5.3)	16 (11.9)	2.43 (0.85-6.97)			
	Dominant	A/A	70 (73.7)	92 (68.7)	1.00	0.41	314.1	321
		A/G-G/G	25 (26.3)	42 (31.3)	1.28 (0.71-2.29)			
	Recessive	A/A-A/G	90 (94.7)	118 (88.1)	1.00	0.075	311.6	318.5
		G/G	5 (5.3)	16 (11.9)	2.44 (0.86-6.91)			
	Overdominant	A/A-G/G	75 (79.0)	108 (80.6)	1.00	0.76	314.7	321.6
		A/G	20 (21.1)	26 (19.4)	0.90 (0.47-1.73)			
*IFIH1* rs1990760	Codominant	T/T	45 (47.4)	69 (41.8)	1.00	0.66	346.5	357.2
		C/T	40 (42.1)	75 (45.5)	1.22 (0.71-2.09)			
		C/C	10 (10.5)	21 (12.7)	1.37 (0.59-3.18)			
	Dominant	T/T	45 (47.4)	69 (41.8)	1.00	0.39	344.6	351.7
		C/T-C/C	50 (52.6)	96 (58.2)	1.25 (0.75-2.08)			
	Recessive	T/T-C/T	85 (89.5)	144 (87.3)	1.00	0.60	345.1	352.2
		C/C	10 (10.5)	21 (12.7)	1.24 (0.56-2.76)			
	Overdominant	T/T-C/C	55 (57.9)	90 (54.5)	1.00	0.60	345.1	352.2
		C/T	40 (42.1)	75 (45.5)	1.15 (0.69-1.91)			
*DDX58* rs73479410	Codominant	G/G	56 (59.0)	103 (62.4)	1.00	0.56	346.2	356.9
		A/G	32 (33.7)	55 (33.3)	0.93 (0.54-1.61)			
		A/A	7 (7.4)	7 (4.2)	0.54 (0.18-1.63)			
	Dominant	G/G	56 (59.0)	103 (62.4)	1.00	0.58	345	352.2
		A/G-A/A	39 (41.0)	62 (37.6)	0.86 (0.52-1.45)			
	Recessive	G/G-A/G	88 (92.6)	158 (95.8)	1.00	0.29	344.2	351.4
		A/A	7 (7.4)	7 (4.2)	0.56 (0.19-1.64)			
	Overdominant	G/G-A/A	63 (66.3)	110 (66.7)	1.00	0.95	345.4	352.5
		A/G	32 (33.7)	55 (33.3)	0.98 (0.58-1.68)			

n, the number of examined COVID-19 patients (cases) and healthy individuals (controls); OR, odds ratio; 95% CI, 95% confidence interval; *p*, logistic regression model; *p_B_*, the significance level after Bonferroni’s correction for multiple testing; *p_B_* was 0.00625 (raw *p*-value/8); AIC, Akaike Information Criterion; BIC, Bayesian Information Criterion(https://snpstats.net). A significant *p*-value is highlighted in bold.

Linkage disequilibrium (LD) analysis demonstrated that the *TLR8* rs3764879 and rs3764880 SNPs exhibited a high level of disequilibrium in the control group (D′ = 1, r^2^ = 1; **Supplementary Figure 1A**). Although D′ indicated strong linkage disequilibrium between the *TLR3* rs3775290 and rs3775291 SNPs, the relatively low r² value suggests limited correlation due to differences in allele frequencies (D′ = 1, r^2^ = 0.184; **Supplementary Figure 1B**). Moreover, LD analysis revealed that the *TLR8* rs3764879 and rs3764880 SNPs exhibited a high level of disequilibrium (D’ = 0.883, r^2^ = 0.729), whereas weak LD was noted among *TLR3* SNPs (D’ = 0.175, r^2^ = 0.006) in the study group (**Supplementary Figures 1C, D**). Haplotype association analysis identified that the common CA haplotype within the *TLR8* gene (comprising rs3764879 and rs376880 SNPs) was significantly associated with a reduced risk of COVID-19 hospitalization (OR = 0.57; *p* = 0.0215; **Supplementary Table 2**).

### 3.4 Associations of *TLR7* rs179008 polymorphism with COVID-19 severity in the study cohort

Comparisons of genotype distributions were performed to identify potential associations between PRR genetic variants and COVID-19 severity. Although the *TLR7* rs179008 SNP was excluded from genetic analyses, this polymorphism was investigated for its correlation with disease severity. The distribution of genotypes for selected *TLR* and *RLR* SNPs among patients with different disease severities, classified according to the WHO Clinical Progression Scale, is shown in **Table 5**. Among patients with a WHO score 4, 22 (54%) had the *TLR7* (rs179008) A/A genotype, while 19 (46%) carried the A/T or T/T genotype. Among patients with a WHO score ≥ 5, 90 (74%) had the A/A genotype, whereas 31 (26%) carried the A/T or T/T genotype (*p* = 0.0131). The carriers of the T allele (A/T or T/T) were less frequently represented among individuals with more severe disease. Interestingly, the T/T genotype of *TLR7* rs179008 was significantly more prevalent in the control group than in hospitalized females (13.8% vs. 1.4%, *p* = 0.010; **Supplementary Table 1**). No other statistically significant associations were observed between *PRR* SNPs and disease course.

**Table 5:** The distribution of genotypes for selected *TLR*/*RLR* SNPs among patients with different disease severities, classified according to the WHO scale.

Gene	SNP ID	Allele	Genotype	WHO scale		*p*-value
				Score 4	Score ≥ 5	
*TLR3*	rs3775290	C>T	C/C	10 (43%)	45 (45%)	0.9514
			C/T or T/T	13 (57%)	54 (55%)	
	rs3775291	C>T	C/C	23 (56%)	58 (48%)	0.3907
			T/C or TT	18 (44%)	62 (52%)	
	rs3775296	C>A	C/C	18 (75%)	53 (57%)	0.1688
			C/A or A/A	6 (25%)	40 (43%)	
*TLR7*	rs179008	A>T	A/A	22 (54%)	90 (74%)	**0.0131**
			A/T or T/T	19 (46%)	31 (26%)	
	rs3853839	C>G	C/C	17 (68%)	76 (73%)	0.7950
			C/G or G/G	8 (32%)	28 (27%)	
	rs5741880	G>T	G/G	16 (94%)	72 (94%)	1.0000
			G/T	1 (6%)	5 (6%)	
*TLR8*	rs3764879	C>G	C/C	17 (65%)	70 (69%)	0.9355
			C/G or GG	9 (35%)	32 (31%)	
	rs3764880	A>G	A/A	17 (65%)	73 (69%)	0.9150
			A/G or G/G	9 (35%)	33 (31%)	
*IFIH1*	rs1990760	C>T	C/C	4 (10%)	17 (14%)	0.5978
			C/T or T/T	37 (90%)	105 (86%)	
*DDX5*	rs73479410	G>A	A/G or A/A	13 (32%)	49 (40%)	0.4360
			G/G	28 (68%)	73 (60%)	

*P*, Pearson’s χ² test, with Yates’s continuity correction or Fisher’s exact test used where necessary. A significant *p*-value is highlighted in bold.

### 3.5 Factors associated with COVID-19 severity – logistic regression model

**Figure 1:**
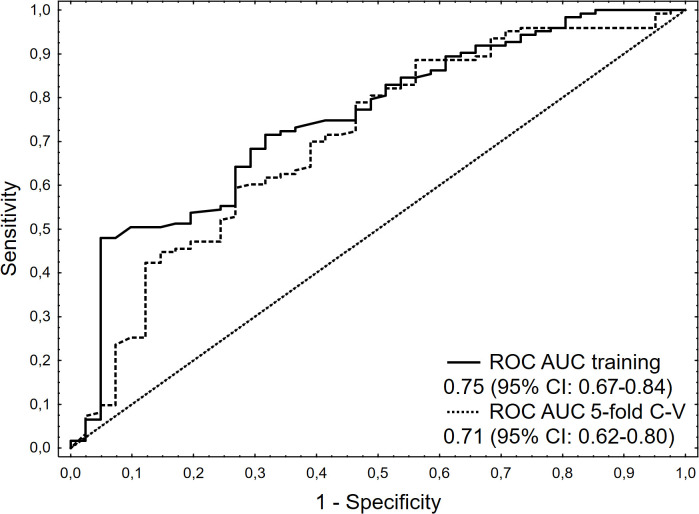
Receiver operating characteristic (ROC) curves of the multivariable logistic regression model for the discrimination of patients with COVID-19 severity grade ≥5 (according to the WHO scale). The solid line represents the ROC curve obtained from the training dataset, while the dashed line indicates performance after 5-fold cross-validation. The area under the curve (AUC) was 0.75 (95% CI: 0.67–0.84) for the training dataset and 0.71 (95% CI: 0.62–0.80) after cross-validation. The diagonal line represents the line of no discrimination.

To assess potential factors influencing COVID-19 severity, including the *TLR7* rs179008 genotype, univariate logistic regression analyses were performed (**Table 6**). Among the analyzed factors, carriage of the T allele of *TLR7* rs179008 (A/T or T/T genotypes) was associated with a reduced risk of severe COVID-19 (OR = 0.40; 95% CI: 0.19–0.83; *p* = 0.0145). Conversely, age between 50 and 69 years (OR = 2.86; 95% CI: 1.20–6.80; *p* = 0.0175) and elevated C-reactive protein (CRP) levels (OR = 1.006; 95% CI: 1.00–1.01; *p* = 0.04) were linked to a worse prognosis. In the final multivariate model, both the A/T and T/T genotypes of *TLR7* rs179008 (OR = 0.46; 95% CI: 0.21–1.00; *p* = 0.0487) and older age (OR = 2.80; 95% CI: 1.14–6.91; *p* = 0.0252) remained statistically significant predictors. The Hosmer–Lemeshow test indicated a good fit of the model (*p* = 0.1832). The model achieved an AUC of 75.1% (95% CI: 66.6–83.6%) on the training dataset. After 5-fold cross-validation, the model maintained its discriminative potential, yielding an AUC of 70.5%, with a sensitivity and specificity at the Youden-optimized cutoff of 78.9% and 53.7%, respectively (**Figure 1**).

**Table 6:** Univariate and multivariate logistic regression analysis of factors associated with higher severity of the COVID-19 course (score ≥5 in the WHO scale).

Variable	Level	*p*-value	OR	95% CI		*p*-value	OR	95% CI	
				Lower	Upper			Lower	Upper
*TLR7* rs179008	A/T or T/T	0.0145	0.399	0.191	0.834	0.0487	0.461	0.214	0.996
	A/A	*Reference*				*Reference*			
Gender	Male	0.4679	1.300	0.640	2.643				
	Female	*Reference*			
Age (years)	50-69	0.0175	2.857	1.201	6.795	0.0252	2.803	1.137	6.913
	>70	0.2007	1.823	0.727	4.573	0.2473	1.764	0.674	4.616
	<50	*Reference*							
CCI*	–	0.0883	1.200	0.973	1.480
CRP*	–	0.0400	1.006	1.000	1.012	0.0883	1.005	0.999	1.011
D-dimer*	–	0.5920	1.000	1.000	1.000				

OR, odds ratio; 95% CI, 95% confidence interval; *p*, logistic regression model and the Hosmer–Lemeshow test; *, variables analyzed as continuous.

### 3.6 Cytokine/chemokine/growth factor profiles in patients with COVID-19 in relation to disease severity and *PRR* genotypes

**Figure 2:**
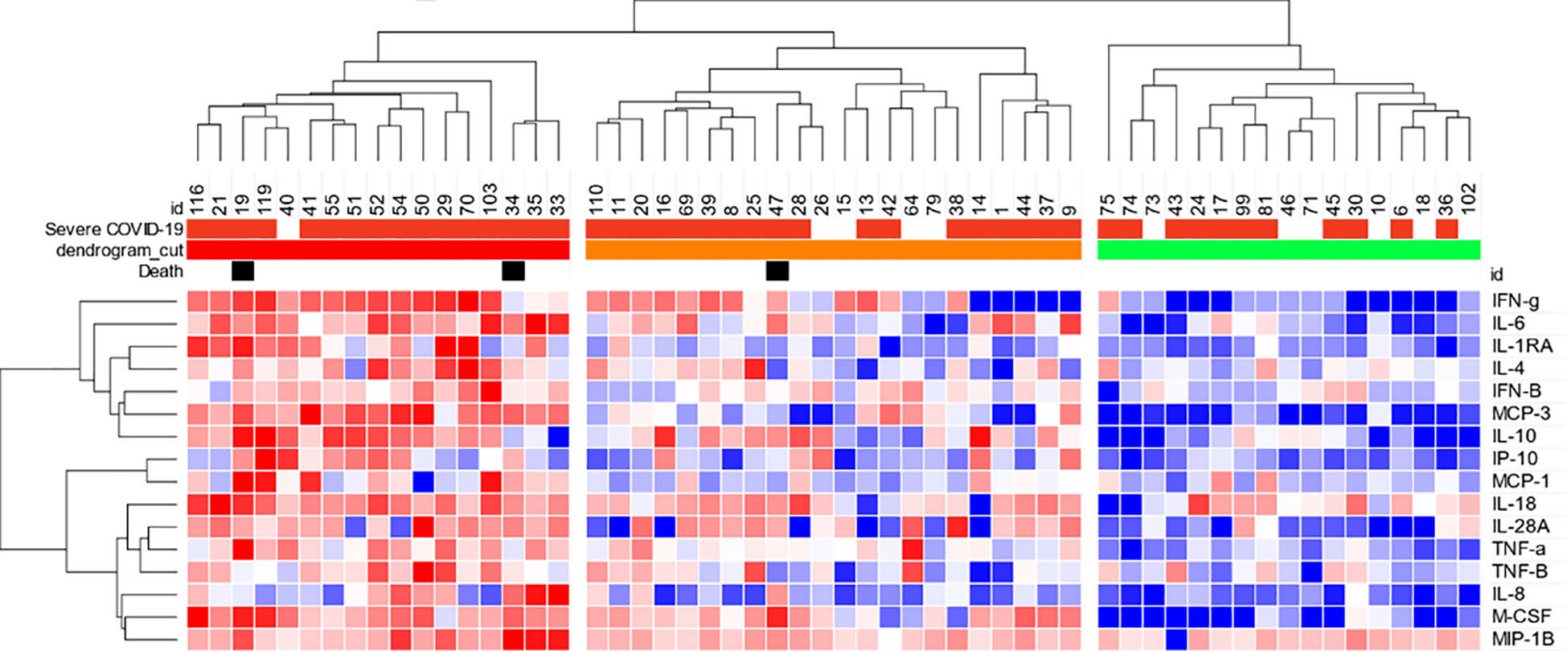
A heatmap visualizing three distinct cytokine/chemokine/factor profile groups—*’*red*’*, *‘*orange*’*, and *‘*green*’*—with an increasing occurrence of non-severe cases across the groups. For heatmap generation, log_2_-transformed values were used, and hierarchical clustering analysis was performed using Euclidean distance and the complete linkage method. Data were visualized using a color scale ranging from blue (lower cytokine concentrations) to red (higher concentrations). Heatmap visualization was performed using Morpheus (Broad Institute; https://software.broadinstitute.org/morpheus).

To investigate potential differences in the inflammatory immune response associated with specific *PRR* SNPs and COVID-19 severity, we compared the serum concentrations of 16 cytokines, chemokines, and growth factors across the respective patient groups. In exploratory clustering analysis (**Figure 2**), we identified three distinct protein-expression clusters: *‘*red*’*, *‘*orange*’*, and *‘*green*’* clusters. A trend toward an increasing frequency of nonsevere cases was observed across these clusters (5.88%, 18.18%, and 35.29%, respectively), although this difference did not reach statistical significance (*p* = 0.0950, χ² test). Additionally, two fatal cases were recorded in the *‘*red*’* cluster, one in the *‘*orange*’* cluster, and none in the *‘*green*’* cluster. In patients with severe COVID-19 (score ≥ 6), significantly higher concentrations of eight proteins were detected: IL-4 (median 3.25 pg/mL, IQR: 1.94–6.13 vs. 1.92 pg/mL, IQR: 1.03–2.32; *p* = 0.0312), IL-6 (median 11.53 pg/mL, IQR: 2.41–54.86 vs. 1.12 pg/mL, IQR: 0.08–2.80; *p* = 0.0038), IL-8 (median 26.40 pg/mL, IQR: 10.00–92.10 vs. 14.33 pg/mL, IQR: 2.77–39.14; *p* = 0.0373), IL-18 (median 51.62 pg/mL, IQR: 35.18–87.13 vs. 15.95 pg/mL, IQR: 8.26–28.94; *p* = 0.0008), M-CSF (median 126.76 pg/mL, IQR: 37.85–201.58 vs. 22.78 pg/mL, IQR: 3.48–83.15; *p* = 0.0247), MIP-1β (median 94.14 pg/mL, IQR: 59.09–120.46 vs. 63.20 pg/mL, IQR: 45.59–87.49; *p* = 0.0328), TNF-α (median 42.62 pg/mL, IQR: 31.82–55.40 vs. 22.17 pg/mL, IQR: 12.15–39.19; *p* = 0.0444), and TNF-β (median 33.97 pg/mL, IQR: 19.57–49.47 vs. 16.97 pg/mL, IQR: 8.73–21.53; *p* = 0.0227) (**Table 7**).

**Table 7:** The comparison of cytokine/chemokine/factor profiles in patients with severe and moderate COVID-19.

Cytokine	Median severe COVID-19	IQR		Median moderate COVID-19	IQR		*p*-value
		Q1	Q3		Q1	Q3	
IFN-ß	11.34	1.78	22.48	6.60	1.78	39.43	0.9093
IL-28A	64.64	16.77	86.08	42.38	16.77	67.12	0.2756
IFN-γ	54.99	2.60	168.75	2.60	2.60	4.25	0.0582
IL-1RA	4.75	2.66	12.91	2.66	2.66	3.64	0.1292
IL-4	3.25	1.94	6.13	1.92	1.03	2.32	**0.0312**
IL-6	11.53	2.41	54.86	1.12	0.08	2.80	**0.0038**
IL-8	26.40	10.00	92.10	14.33	2.77	39.14	**0.0373**
IL-10	12.33	3.51	29.28	2.86	0.39	12.86	0.0663
IL-18	51.62	35.18	87.13	15.95	8.26	28.94	**0.0008**
IP-10	565.78	293.80	1197.92	282.02	193.99	540.60	0.1077
MCP-1	608.53	482.41	993.20	496.51	416.26	626.00	0.1033
MCP-3	14.16	2.35	53.94	2.17	1.17	20.54	0.0541
M-CSF	126.76	37.85	201.58	22.78	3.48	83.15	**0.0247**
MIP-1ß	94.14	59.09	120.46	63.20	45.59	87.49	**0.0328**
TNF-α	42.62	31.82	55.40	22.17	12.15	39.19	**0.0444**
TNF-ß	33.97	19.57	49.47	16.97	8.73	21.53	**0.0227**

All data are presented in pg/mL. IQR, interquartile range; Q1, first/lower quartile; Q3, third/upper quartile; *p*, Mann-Whitney U test. Significant *p-*values are highlighted in bold.

**Figure 3:**
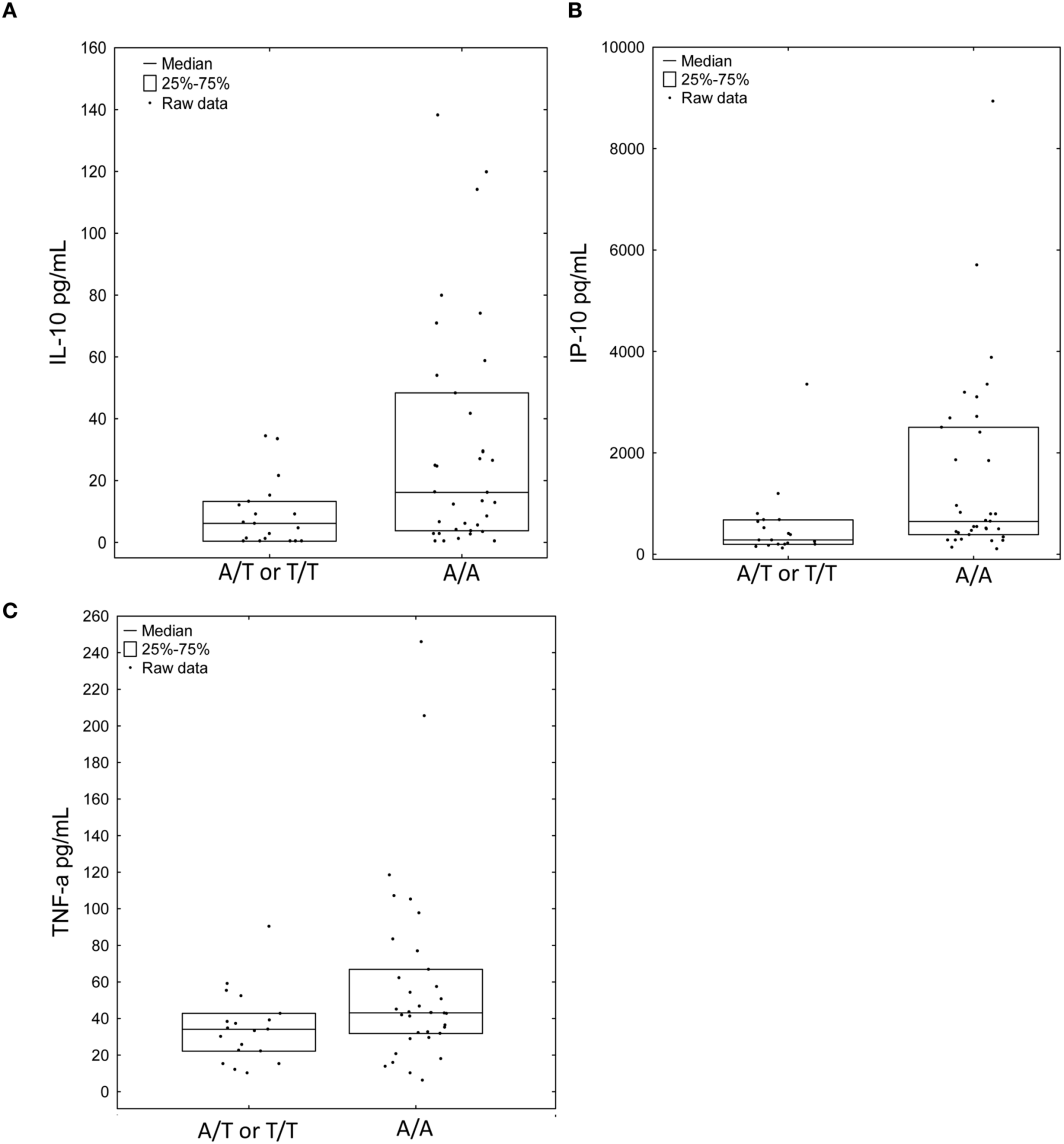
Significant differences in cytokine levels among patients with *TLR7* rs179008 genotypes: **(A)** IL-10, **(B)** IP-10, and **(C)** TNF-α. *P*, Mann-Whitney U test; *p* = 0.0158 **(A)**, *p* = 0.0087 **(B)**, and *p* = 0.0498 **(C)**.

The concentrations of several cytokines and chemokines were significantly altered in patients with COVID-19 with specific *PRR* genotypes. A distinct cytokine profile was identified in patients with the *TLR7* (rs179008) A/T or T/T genotype compared with those with the A/A genotype (**Supplementary Table 3**). Specifically, compared with A/A homozygotes, carriers of at least one T allele (A/T or T/T genotype) exhibited significantly lower concentrations of IL-10 (median 6.14 pg/mL, IQR: 0.39–13.25 vs. 16.15 pg/mL, IQR: 3.76–48.34; *p* = 0.0158), IP-10 (median 282.02 pg/mL, IQR: 195.65–677.48 vs. 646.37 pg/mL, IQR: 386.06–2504.04; *p* = 0.0087), and TNF-α (median 34.10 pg/mL, IQR: 22.17–42.80 vs. 43.07 pg/mL, IQR: 31.82–66.89; *p* = 0.0498) (**Figure 3**). Additionally, a polymorphism in the *DDX58* gene (rs73479410) was associated with higher concentrations of IL-4 (*p* = 0.0076), MCP-1 (*p* = 0.0100), TNF-β (*p* = 0.0445), and IL-18 (*p* = 0.0464). In contrast, *IFIH1* rs1990760, *TLR3* rs3775290, and *TLR8* rs3764879 SNPs were associated with lower serum concentrations of IL-8 (*p* = 0.0061), MCP-3 (*p* = 0.0171), and IL-10 (*p* = 0.0455), respectively.

## 4 Discussion

Genetic polymorphisms in *TLR* genes may lead to dysregulation of TLR-related signaling pathways and receptor expression that may be associated with the development and severity of COVID-19. Moreover, it is still unclear why some individuals develop mild disease, whereas others require hospitalization, especially during the first waves of the COVID-19 pandemic. TLR3- and TLR7-dependent IFN-I production by respiratory epithelial cells and plasmacytoid dendritic cells, respectively, is essential for host defense during the early phase of SARS-CoV-2 infection (50). While early TLR7-dependent IFN-I responses are indispensable for viral control, excessive or prolonged activation of this pathway may promote hyperinflammatory responses that contribute to tissue damage and disease progression. Our findings indicate that among hospitalized patients with COVID-19, carriers of the T allele of the *TLR7* rs179008 SNP develop lower disease severity and lower serum cytokine levels. The *TLR7* rs3853839 polymorphism may be associated with increased risk of hospitalization due to COVID-19.

The *TLR7* gene is located on the short arm of the X chromosome (p22.2) and encodes an endosomal innate immune sensor that recognizes uridine-containing ssRNAs of viral origin or guanosine analogs (51). Upon ligand binding, TLR7 undergoes dimerization, leading to the recruitment of the TIR-containing adaptor MYD88. This results in the formation of signaling complexes involving IRAK1/4 and TRAF3/6, leading to the activation of the transcription factors NF-κB and IRF3/7, which induce proinflammatory cytokines and IFNs, respectively (34). TLR7 is a crucial sensor in SARS-CoV-2 infection and has been associated with COVID-19 susceptibility and severity (20, 22, 25, 33, 34, 52). It has been proposed that the gene dosage of *TLR7* may partly explain the differences in the sex-biased severity observed in patients with COVID-19 (53). Hence, *TLR7* expression profiles may cause sex-based differences in immune responses, causing females to respond more actively to single-stranded viruses (54). Male patients with reduced *TLR7*-related gene expression demonstrate an impaired IFN responses and are predisposed to severe COVID-19 (22, 34). Sex differences in the TLR7-mediated response were also observed in other viral diseases, including higher viral loads in men than in women with early HIV infection (55). Given that the *TLR7* gene is encoded on the X chromosome, males are fully exposed to the functional consequences of disadvantageous variants, whereas females may partially compensate through biallelic expression or X-chromosome mosaicism. Genetically determined variation in TLR7 signaling might contribute to the well-documented male bias in severe COVID-19 outcomes. *TLR7* variants have been reported to be genetic risk factors for COVID-19 susceptibility, severity, and long-term complications (56, 57).

In our cohort, the homozygous GG genotype and G allele of *TLR7* rs3853839 C>G occurred more frequently in hospitalized patients with COVID-19 than in sex- and age-matched controls. However, multivariate logistic regression analysis revealed that this polymorphism was not significantly associated with disease severity or cytokine expression among hospitalized patients. Previously, the rs3853839 polymorphism was identified as a factor associated with severe COVID-19 and poor clinical outcomes in the Egyptian population (31). Similarly, a Spanish study identified this polymorphism as a risk factor for predicting increased disease severity (32).El-Hefnawy et al. reported that *TLR7* rs3853839 SNP was also associated with elevated *TLR7* mRNA expression levels in COVID-19 patients (31). The rs3853839 polymorphism is located in the 3’ untranslated region (3’ UTR) of *TLR7* gene and affects *TLR7* RNA turnover (58, 59). It has been previously associated with increased expression of *TLR7* mRNA and protein, as well as the upregulation of IFN-stimulated genes (ISGs) in patients with systemic lupus erythematosus (SLE) (58, 60). Altered *TLR7* expression levels are implicated in several chronic disorders, indicating a key role for this receptor in modulating inflammation (51, 61). Increased *TLR7* expression associated with the rs3853839 G allele may predispose individuals to excessive inflammatory responses, particularly in the later stages of infection, when dysregulated IFN signaling has been linked to immunopathology rather than viral control. These results indicated that the *TLR7* rs3853839 SNP may play a role in COVID-19 development in different ethnic groups.

In this study, another polymorphism in the *TLR7* gene, the rs179008 SNP, was associated with a decreased risk of severe COVID-19 and lower serum concentrations of several cytokines, including IP-10, IL-10, and, in a weaker manner, TNF-α. The presence of at least one recessive allele (A/T or T/T genotype) decreased the risk of severe COVID-19. Similarly, the T allele of rs179008 was found to be protective against COVID-19 disease progression in a small sample size study in the Iraqi population cohort (62). Few other studies have investigated the association of this polymorphism with SARS-CoV-2 infection and COVID-19 development. The heterozygous A/T genotype and T allele were linked to a highly increased risk of susceptibility to SARS-CoV-2 infection among the Iranian population (30). The rs179008 polymorphism was also associated with an increased risk of COVID-19 pneumonia (63) and recurrent COVID-19 (64) in other populations. However, no significant association was found between the *TLR7* rs179008 polymorphism and the outcome of COVID-19 pneumonia (63). Alseoudy et al. reported a lower frequency of the minor T allele for this polymorphism compared to other populations, especially Europeans (63). The differences in the results of SNP association in different population groups could be explained by unique genetic backgrounds and environmental factors. This nonsynonymous polymorphism within exon 3 promotes an amino acid substitution, from glutamine to leucine at residue 11 (Q11L), in the structure of TLR7. The minor T allele has been associated with reduced *TLR7* expression and impaired receptor signaling (65). The missense variant alters TLR7 functionality and intracellular signaling in several viral infections, including SARS-CoV-2 (66), HIV (67), and HCV (68). Because TLR7 recognizes conserved features of viral ssRNA, the observed associations are likely driven by host-intrinsic immune mechanisms rather than variant-specific viral properties. Notably, IP-10 emerged as a convergent biomarker linking *TLR7* genetic variation with clinical severity. An association of the missense variant with lower IP-10 levels in patients with other viral infections was observed. Among women from a French cohort study of acute HIV-1 patients, carriage of the T allele rs179008 was associated with lower IP-10 plasma concentrations and viremia (65). During HIV infection, IP-10 is known to display a robust positive correlation with viral load, dissemination, and the establishment of a viral reservoir. In COVID-19 patients, IP-10 was selected as one of the key biomarkers associated with the severity and risk of critical disease (69, 70).

The *TLR3* gene consists of five exons and is located on human chromosome 4q35.1. *TLR3* polymorphisms affect the expression and cell surface localization of TLR3 and influence the induction of the NF-κB cascade (71). Inborn errors in TLR3- and IRF7-dependent IFN-I immunity have been reported to underlie life-threatening COVID-19 pneumonia in patients with no prior severe infection (18). Both the *TLR3* rs3775290 and the *TLR3* rs3775291 SNPs are synonymous and nonsynonymous mutations in exon 4, respectively. *TLR3* rs3775291 SNP changes the amino acids from leucine to phenylalanine at residue 412 (L412F). The polymorphism rs3775296, located in the 5′-UTR of *TLR3*, has been reported to be associated with elevated *TLR3* expression and other diseases, including SLE and preeclampsia (72, 73). In the Turkish population, the TT genotype of the *TLR3* rs3775290 polymorphism was found to be more frequent in COVID-19 patients than in healthy controls, an association that was specifically observed in males (36). The TT genotype and T allele of *TLR3* rs3775290 were linked to an increased risk of COVID-19 pneumonia and increased mortality in the Egyptian population (63). The L412F mutation was also associated with the risk of COVID-19 in the Italian population (74). *TLR3* rs3775291 and rs3775296 were not significantly associated with COVID-19 in the Iranian population (30). Moreover, the recessive allele and heterozygous genotype of the *TLR3* rs3775296 polymorphism have been proposed as protective factors against COVID-19 (36). In the present study, no significant associations were found for any of the studied *TLR3* SNPs with COVID-19 development or severity. Similar results were reported by other groups (75, 76).

The *IFIH1* gene is located on the reverse strand of chromosome 2 and encodes the MDA5 helicase. The *IFIH1* rs1990760 polymorphism (c.2836G>A, A946T) encodes a gene variant related to different susceptibilities to viral infections and autoimmune disorders. The rs1990760 SNP is associated with decreased inflammation and better prognosis in patients with COVID-19 (41). In contrast, no significant association was found between the rs1990760 SNP and COVID-19 severity in the Kermanshah population (38). *DDX58* gene expression decreased with increasing number of minor alleles of rs73479410 (37). In our study, no significant association was found between the *IFIH1* rs1990760 or *DDX58* rs73479410 polymorphisms and COVID-19 development or severity. The inconsistencies observed in association studies likely stem from insufficient statistical power. This is often driven by limitations in sample size, the use of stringent adjustments for multiple testing, low minor allele frequencies, and various population-dependent factors. Additionally, the specific genotyping methods employed may account for differences in results across studies. In this study, most *PRR* polymorphisms were genotyped using TaqMan allelic discrimination assays due to their high efficiency and accuracy. The *TLR3* rs3775296 variants were identified using the PCR-RFLP method, which provided a reliable and cost-effective alternative. The selection of this method was based on the structural characteristics of the SNP site and the availability of a compatible restriction enzyme.

The strengths of this study are the identification of genetic risk factors associated with COVID-19 severity, and their impact on serum cytokine concentrations. The vast majority of the studied cases (86%) were from the Alpha wave of SARS-CoV-2, while the remaining patients were infected with SARS-CoV-2 during the Omicron wave. To exclude population differences, all cases from the study and control groups were collected in the same city and were ethnically matched. Like other genetic association studies, this study has several limitations. First, this study may be limited by the relatively small number of cases, although we enrolled COVID-19 patients who were clinically verified by experienced clinicians. Since the sample size was relatively small, this may have limited the statistical power. Second, the case group of patients hospitalized for COVID-19 was relatively heterogeneous, including patients whose disease severity ranged from 4 to 10 according to the WHO scale. Third, our analysis included both men and women for genes located on the X chromosome, which may introduce sex-related biases. Another limitation of this study is its case–control design, which primarily assesses genetic factors associated with SARS-CoV-2 susceptibility. While valuable, these comparisons are susceptible to several confounding variables, such as differences in exposure risk or the potential misclassification of individuals in the control group. To address this, we conducted additional analyses focused on the COVID-19 cohort, including genotype distribution across disease severity levels and logistic regression modeling. Such approaches offer a more direct evaluation of the role of host genetic factors in the clinical progression of COVID-19. Therefore, while our findings suggest that specific SNPs may contribute to viral susceptibility, these results should be interpreted with caution. In contrast, the genetic associations identified within the infected population, specifically the *TLR7* rs179008 polymorphism, likely provide a more accurate and direct reflection of the genetic drivers behind COVID-19 severity. Further studies comparing patients with asymptomatic or mild SARS-CoV-2 infection to those with severe COVID-19 will provide critical insights into the genetic factors that determine clinical outcomes.

In future studies, we recommend using larger sample sizes to confirm our findings, as well as including functional assays and longitudinal immune profiling. The proposed study involves analyzing gene expression profiles in peripheral blood mononuclear cells isolated from carriers of *TLR7* variants and cells transfected with these functional variants following stimulation with imiquimod, a known TLR7 agonist. An understanding of the molecular mechanisms underlying PRR functionality is critical, as these insights may provide an understanding of the causes of COVID-19 susceptibility.

## Conclusions

The results of the present study suggest that the rs3853839 C>G polymorphism in the *TLR7* gene may be associated with an increased risk of moderate or severe COVID-19 that requires hospitalization. Our results also suggest an association between the *TLR7* rs179008 SNP and the severity or lower expression of serum cytokine concentrations among hospitalized patients with COVID-19. Hence, we propose that this variant of the *TLR7* gene may be a factor for decreased severity of COVID-19. No significant relationships were found between the other examined SNPs and COVID-19.

## Data Availability

The raw data supporting the conclusions of this article will be made available by the authors, without undue reservation. The datasets generated and analyzed for this study will be available in the RepOD [https://repod.icm.edu.pl/dataset.xhtml?persistentId=doi:10.18150/NA2YBO].
