# Supplementary material for: TLR7 polymorphisms are associated with COVID-19 susceptibility and severity
Source: Front Immunol. 2026 May 28;17:1837208. doi: 10.3389/fimmu.2026.1837208 (PMC13253635; doi:10.3389/fimmu.2026.1837208)
Supplement: Supplementary file 1 [file DataSheet1.pdf]

Supplementary Material

1 Supplementary Tables and Figures

1.1 Supplementary Tables

Supplementary Table 3. Frequencies of *TLR3*, *TLR7*, *TLR8*, *IFIH1*, and *DDX58* SNPs genotypes and alleles in healthy volunteers (controls) and women with COVID-19 (cases).

Gene	SNP ID	Genotype allele	Genotype/allele frequencies, n (%) controls	cases	*p*-value
TLR3	rs3775290	CC	46 (48.4)	30 (52.6)	0.738
		CT	40 (42.1)	17 (29.8)	0.180
		TT	9 (9.5)	10 (17.5)	0.229
		C	132 (69.5)	77 (67.5)	0.823
		T	58 (30.5)	37 (32.5)	
	rs3775291	CC	49 (51.6)	37 (52.1)	0.946
		CT	36 (37.9)	26 (36.6)	0.995
		TT	10 (10.5)	8 (11.3)	0.879
		C	134 (70.5)	100 (70.4)	0.983
		T	56 (29.5)	42 (29.6)	
	rs3775296	CC	60 (71.4)	31 (56.4)	0.100
		CA	24 (28.6)	21 (38.2)	0.318
		AA	0 (0)	3 (5.5)	0.117
		C	144 (85.7)	83 (75.5)	**0.045**
		A	24 (14.3)	27 (24.5)	
TLR7	rs179008	AA	56 (59.6)	41 (56.9)	0.856
		AT	25 (26.6)	30 (41.7)	0.060
		TT	13 (13.8)	1 (1.4)	**0.010**
		A	137 (72.9)	112 (77.8)	0.371
		T	51 (27.1)	32 (22.2)	
	rs3853839	CC	77 (81.0)	43 (70.5)	0.183
		CG	15 (15.8)	11 (18.0)	0.883
		GG	3 (3.2)	7 (11.5)	0.083
		C	169 (88.9)	97 (79.5)	**0.033**
		G	21 (11.1)	25 (20.5)	
	rs5741880	GG	80 (87.9)	40 (93.0)	0.548
		GT	11 (12.1)	3 (7.0)	
		G	171 (94.0)	83 (96.5)	0.559

		T	11 (6.0)	3 (3.5)	
TLR8	rs3764879	CC	70 (73.7)	38 (62.3)	0.185
		CG	20 (21.1)	14 (23.0)	0.935
		GG	5 (5.3)	9 (14.8)	0.082
		C	160 (84.2)	90 (73.8)	**0.035**
		G	30 (15.8)	32 (26.2)	
	rs3764880	AA	70 (73.7)	39 (62.9)	0.209
		AG	20 (21.1)	14 (22.6)	0.977
		GG	5 (5.3)	9 (14.5)	0.089
		A	160 (84.2)	92 (74.2)	**0.042**
		G	30 (15.8)	32 (25.8)	
IFIH1	rs1990760	CC	10 (10.5)	7 (9.9)	0.888
		CT	40 (42.1)	40 (56.3)	0.097
		TT	45 (47.4)	24 (33.8)	0.111
		C	60 (31.6)	54 (38.0)	0.268
		T	130 (68.4)	88 (62.0)	
DDX58	rs73479410	GG	56 (59.0)	49 (68.1)	0.296
		AG	32 (33.7)	20 (27.8)	0.517
		AA	7 (7.4)	3 (4.2)	0.593
		G	144 (75.8)	118 (81.9)	0.222
		A	46 (24.2)	26 (18.1)	

n, number of cases; *p*, χ^2 test with Yates' correction. Significant *p*-values are highlighted in bold.

Supplementary Table 2. Association of haplotypes and COVID-19 hospitalization.

Block	Haplotype	Frequency	Case / control frequency	OR	Pearson's χ^2	*p*-value
TLR7	GA	0.714	0.736 / 0.682	1.30	1.573	0.2098
	GT	0.241	0.228 / 0.258	0.85	0.534	0.465
	TA	0.039	0.033 / 0.047	0.69	0.594	0.441
TLR8	CA[a]	0.790	0.753 / 0.842	0.57	5.286	**0.0215**
	GG	0.181	0.197 / 0.158	1.31	1.17	0.2794
	GA[b]	0.018	0.031 / 0.0	NE	5.812	**0.0159**
	CG	0.011	0.019 / 0.0	NE	3.611	0.0574

OR, odds ratio; [a] protective haplotype; [b] risk haplotype; block of *TLR7* SNPs (order of SNPs: rs5741880 and rs179008); block of *TLR8* SNPs (order of SNPs: rs3764879 and rs3764880); NE, not estimable; *p*, Pearson's χ^2 test, Haploview software. A significant *p*-value is highlighted in bold.

Supplementary Table 3. Comparison of cytokine/chemokine/factor profiles between patients with different *TLR7* rs179008 genotypes. All data are presented in pg/mL.

**Protein**	**Median *TLR7* rs179008 A/T or T/T**	**IQR**		**Median *TLR7* rs179008 A/A**	**IQR**		***p*-value**
		**Q1**	**Q3**		**Q1**	**Q3**	
IFN-ß	8.58	1.78	27.22	11.34	2.69	22.85	0.4722
IL-28A	42.38	16.77	75.75	64.64	16.77	92.37	0.2564
IFN-γ	13.98	2.60	123.81	54.99	2.60	170.38	0.4722
IL-1RA	2.66	2.66	7.86	4.72	2.66	12.91	0.3880
IL-4	2.05	1.67	4.52	3.24	1.73	6.44	0.2203
IL-6	2.41	1.49	12.43	12.82	1.91	54.86	0.1591
IL-8	18.27	7.45	160.52	20.12	9.59	82.80	0.7062
IL-10	6.14	0.39	13.25	16.15	3.76	48.34	**0.0158**
IL-18	28.94	8.26	49.93	51.62	26.37	89.47	0.0543
IP-10	282.02	195.65	677.48	646.37	386.06	2504.04	**0.0087**
MCP-1	553.30	493.06	862.72	596.27	444.61	1014.81	0.8295
MCP-3	3.81	1.17	53.67	16.18	5.19	53.31	0.0853
M-CSF	60.12	3.48	134.98	126.76	22.78	201.58	0.1434
MIP-1ß	75.96	57.79	100.40	94.14	50.92	120.46	0.6406
TNF-α	34.10	22.17	42.80	43.07	31.82	66.89	**0.0498**
TNF-ß	21.53	11.14	49.47	33.97	16.65	47.98	0.3223

IQR, the interquartile range; Q1, the first quartile (also called the lower quartile); Q3, the third quartile (also called the upper quartile); *p*, Mann-Whitney U test. Significant *p*-values are highlighted in bold.

1.2 Supplementary Figure 1

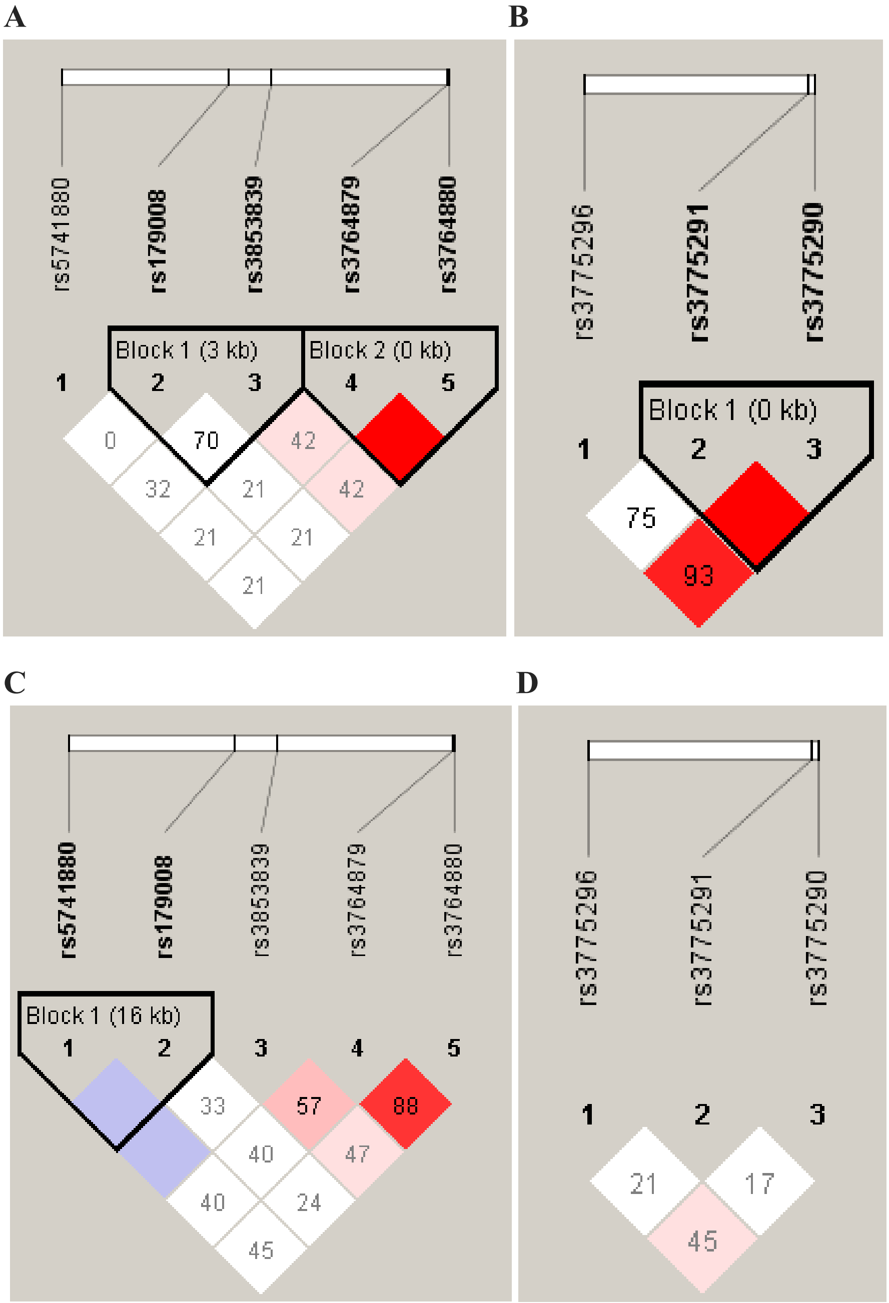

Supplementary Figure 1. Linkage disequilibrium (LD) analysis results of *TLR3*, *TLR7*, and *TLR8* gene variants. *TLR7* (rs179008, rs3853839, and rs741880) and *TLR8* (rs3764879 and rs3764880) polymorphisms in the control (A) and cases (C) groups. *TLR3* (rs3775290, rs3775291, and rs3775296) polymorphisms in the control (B) and cases (D) groups. The numbers in the squares refer to D′ values [multiplied by 100 (|D′| × 100)] of the given pairs of SNPs. The colours represent the relative D' score, where bright red is D' = 1 and shades of pink D' < 1. Empty red squares represent perfect LD (D′ = 1). Bolded triangles reveal haplotype blocks identified using the solid spine of the LD method. LD analysis was performed using Haploview software.
